# Bee Community of Commercial Potato Fields in Michigan and *Bombus impatiens* Visitation to Neonicotinoid-Treated Potato Plants

**DOI:** 10.3390/insects8010030

**Published:** 2017-03-09

**Authors:** Amanda L. Buchanan, Jason Gibbs, Lidia Komondy, Zsofia Szendrei

**Affiliations:** Department of Entomology, Michigan State University, East Lansing, MI 48824, USA; a.lynnbuchanan@gmail.com (A.L.B.); Jason.Gibbs@umanitoba.ca (J.G.); lkomondy@msu.edu (L.K.)

**Keywords:** blue vane trap, bowl trap, *Lasioglossum* spp., *Bombus* spp., *Solanum tuberosum*

## Abstract

We conducted a bee survey in neonicotinoid-treated commercial potato fields using bowl and vane traps in the 2016 growing season. Traps were placed outside the fields, at the field edges, and 10 and 30 m into the fields. We collected 756 bees representing 58 species, with *Lasioglossum* spp. comprising 73% of all captured bees. We found seven *Bombus* spp., of which *B. impatiens* was the only known visitor of potato flowers in our region. The majority of the bees (68%) were collected at the field edges and in the field margins. Blue vane traps caught almost four-times as many bees and collected 30% more species compared to bowl traps. Bee communities did not differ across trap locations but they were different among trap types. We tested *B. impatiens* visitation to neonicotinoid treated and untreated potato flowers in field enclosures. The amount of time bees spent at flowers and the duration of visits were not significantly different between the two treatments. Our results demonstrate that a diverse assemblage of bees is associated with an agroecosystem dominated by potatoes despite the apparent lack of pollinator resources provided by the crop. We found no difference in *B. impatiens* foraging behavior on neonicotinoid-treated compared to untreated plants.

## 1. Introduction

Potato (*Solanum tuberosum* L., Solanaceae) is the fourth main food-crop in the world after corn, wheat and rice [1], but is the only one of these that is not wind pollinated. It is the leading vegetable crop in the United States in terms of production area and farm-gate value [2]. Pollinators are not required for its commercial production because harvested tubers are vegetative plant parts and the plants are effectively propagated vegetatively. Similarly to many other members in the Solanaceae, it has prominent flowers during about two weeks of its growing season. Flower petals can be various shades of white, purple, pink or blue, and bright yellow cone-shaped anthers in the middle of the flower release pollen when vibrated [3]. Multiple flowers are arranged in inflorescences and flowering plants emit large amounts of methyl phenylacetate, which has a sweet floral fragrance [4]. Since potato flowers do not produce nectar, they attract pollen-collecting insects [5]. A few species of bees (Apoidea: Anthophila) have been recorded visiting potato flowers [3,5,6,7,8,9] but information on bees that can be found in or near potato fields is generally lacking.

Potatoes are grown in large-scale monocultures that lack diverse resources for pollinators [10]. Despite agricultural landscape simplification, about 50–60 species of pollinators have been collected from soybean and corn monocultures, indicating that pollinators use and persist in these agroecosystems [11,12,13]. Potato fields produce temporary flushes of flowers that may attract species of polylectic native bees nesting in nearby undisturbed areas. Native bee conservation efforts require surveys and identification of the pollinator composition found in and near under-sampled regions such as potato fields. The identification of rare bee species, for example, could warrant further detailed research into their use of potatoes that could eventually lead to changes in crop management efforts.

Potatoes, like corn and soybean, are produced commercially with neonicotinoid insecticides to control several arthropod pests [14]. This group of systemic insecticides can persist for weeks in plant tissue and is translocated to flowers [15]. The main neonicotinoid used in Michigan commercial potatoes is imidacloprid, applied as an at-planting drench application to >80% of the acreage in the state [16]. Imidacloprid is often cited as the most toxic of the neonicotinoids to bees and it can readily translocate from seed treatment into nectar and pollen [17,18]. Neonicotinoids can often be detected in pollen samples taken from field collected bees; therefore, contact exposure and oral ingestion can both play key roles in causing lethal and sublethal effects in bees [17,18].

Bumblebees (Apidae: *Bombus*) are one of the most common and abundant buzz-pollinators in potato growing regions [5], thus they are likely at a risk of pesticide exposure. Although not studied in potato specifically, pesticides can affect bumblebee physiology and behavior. For example, bumblebee foraging behavior was altered by chronic exposure to a neonicotinoid insecticide [19,20] and their fecundity and colony growth was also negatively impacted by neonicotinoid consumption [21,22,23]. While these studies suggest bees may be negatively affected by neonicotinoids, other work suggests that realistic exposures to neonicotinoids in the field do not negatively affect bees and that sublethal effects do not necessarily result in lasting colony effects, especially if pesticide-free alternative forage is available [24,25,26,27,28,29].

Our goals in this study were to (1) survey and identify the bee community in commercial, neonicotinoid-treated potato fields in Michigan and; (2) evaluate the effect of neonicotinoid (imidacloprid) treatment on bumblebee visitation to potato flowers in field enclosures.

## 2. Materials and Methods

### 2.1. Bee Community Survey in Neonicotinoid-Treated Commercial Potato Fields

#### 2.1.1. Data Collection

**Figure 1:**
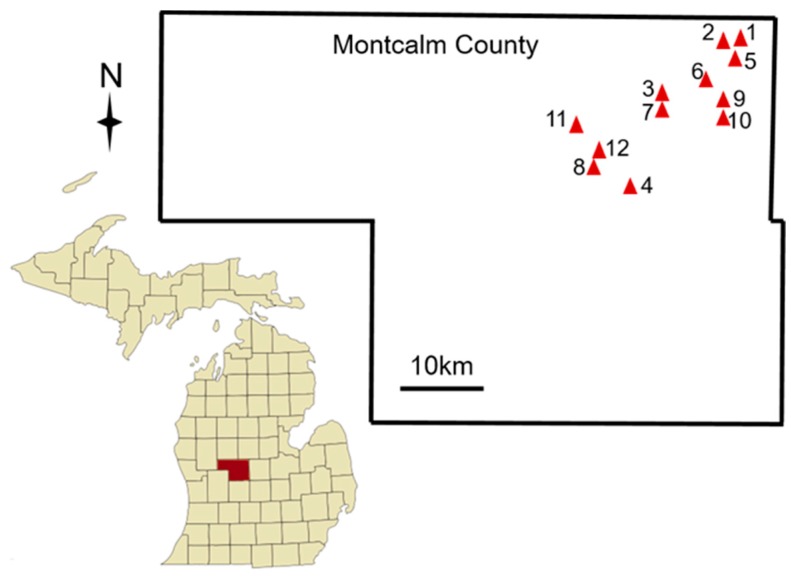
Commercial potato fields were surveyed for bees in Montcalm County (shown in red on the state map), Michigan, USA. Red triangles represent the locations of 12 commercial potato fields, which were used to survey bees. Fields were 0.8–18.2 km apart, with an average of 3.6 km distance between sites.

Bees in commercial potato fields were surveyed using bowl and blue vane traps [30,31] in 12 potato fields (‘sites’) from 21 June to 22 July 2016. Site elevation was 255 m, daily average maximum temperature was 27 °C, daily average minimum was 13.5 °C, and total precipitation was 2 mm during the sampling period [32]. Seasonal climate information for this area is available in Figure S1. All potato seeds were treated with imidacloprid (0.02 L/ha active ingredient) 5–7 days pre-planting, and foliar insecticides were applied during the growing season (Table S1). Plants were 7–9 weeks post-planting at the time of sampling and all were ware potatoes used for chips (Table 1). Fields were sampled when 40–100% blooms were open. Traps were collected after 48 h in the field. Sites 1–4 were established on 21 June 2016, and traps were deployed on 23 June 2016 and again on 25 June 2016. Sites 5–8 were established 5 July 2016 and traps were deployed on 7 July 2016. Sites 9–12 were established on 18 July 2016 and traps were deployed on 20 July 2016 and again on 22 July 2016. Sites were 0.8–18.2 km apart (average minimum distance between sites = 3.6 km) and 6–42 ha in size (mean size 21 ± 4 ha, Figure 1 and Table 1).

Sites were located in a region where 45% of the land has been converted to agricultural fields, and deciduous hardwoods comprise about 25% of the landscape (primarily maple (*Acer* spp.), oak (*Quercus* spp.), ash (*Fraxinus* spp.), beech (*Fagus* spp.), and hemlock (*Tsuga* spp.); [33]). About 30% of the landscape is either open land or turned into housing developments (urban spaces, open water or wetlands, and treeless meadow areas; [33]). In this region, potato field margins typically contain species of Poaceae (*Poa* spp., *Festuca* spp., *Lolium* spp., *Agrostis* spp.); Fabaceae (*Trifolium* spp.); Plantaginaceae (*Plantago* spp.); Caryophyllaceae (common chickweed, *Stellaria media* L. Vill.; mouse-ear chickweed, *Cerastium fontanum* Baumg.; and white campion, *Silene latifolia* Poir.); Lamiaceae (purple dead-nettle, *Lamium purpureum* L.); Oxalidaceae (wood sorrel, *Oxalis stricta* L.); and Asteraceae (e.g., dandelion *Taraxacum officinale* F.H. Wigg; and other Cichoriae, corn chamomile, *Anthemis arvensis* L.). In addition, several species of Solanaceae (eastern black nightshade, *Solanum ptychanthum* Dunal; climbing nightshade, *S. dulcamara* L.; and Carolina horsenettle, *S. carolinense* L.) are common weed species in the sampled region that are congeners of the cultivated potato.

**Figure 2:**
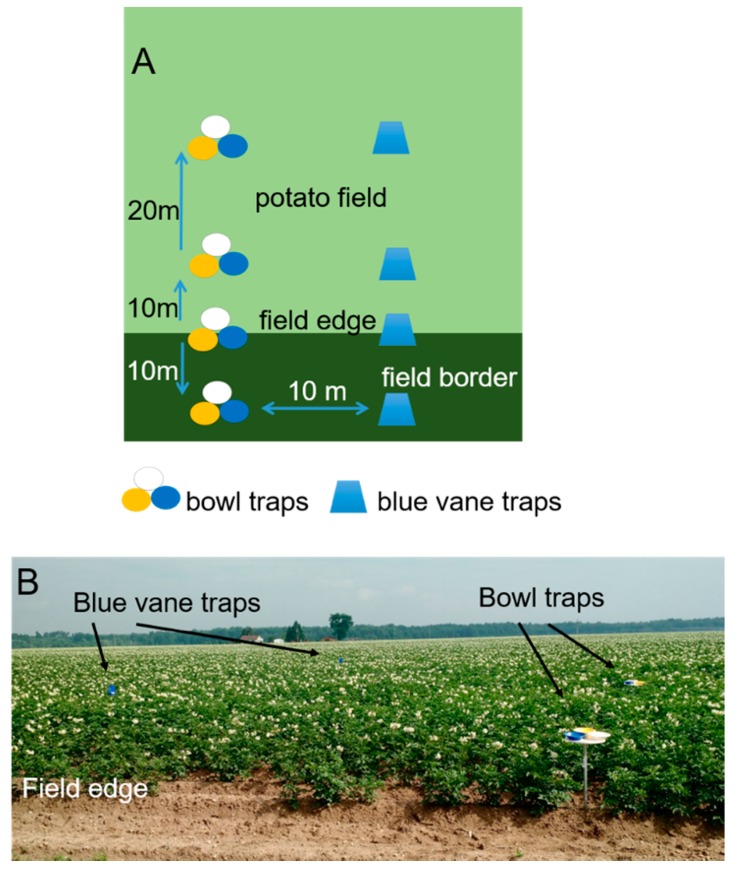
Transects of bowl traps and blue vane traps were placed in each potato field to survey the bee community. Bowl and vane transects were 10 m apart and extended from the field border to 30 m into the field; figure not to scale (**A**); Bowl trap platforms were elevated to canopy height on metal poles; blue vane traps were suspended at canopy height from a metal pole (**B**). Traps were emptied after 48 h.

Each bowl trap consisted of a 40-cm-diameter circular plastic platform elevated to canopy height, on which three plastic bowls (blue #181677, yellow #14260, and white #14258, Party City Corporation, Rockaway, NJ, USA; 19 cm diameter, 0.35 L) were glued. The platforms were attached to the tops of 1-m metal conduits (2 cm diameter) that were pounded into the soil. At the beginning of each 48-h sampling period, bowls were filled to capacity with soapy water (Dawn^®^ dish soap, unscented, colorless, Procter & Gamble, Cincinnati, OH, USA). Each blue vane trap (SpringStar LLC, Woodinville, WA, USA) consisted of a blue vane and 2 L collection container suspended on a metal pole at canopy height. Containers were filled with approximately 1 L of 1:1 propylene glycol:water solution. Traps were set out in transects of four traps with the first located in vegetation outside the potato field (roughly 10 m from the field edge), the second at the potato field edge, the third 10 m into the field, and the fourth 30 m into the potato field (Figure 2). We chose potato field edges that were near mixed hardwoods (about 30 m away from the potatoes). Between the potato field and the woods was a 5–10 m wide strip of mowed grasses and forbs. The average size of wooded areas in this region was 2 km^2^, and the average width of treeless meadow areas adjacent to potato fields was 5–10 m, running the length of the cropped area. Each field had one transect of bowl and one transect of blue vane traps 10 m apart. Bees were collected by pouring the trap solution through a strainer and placing all insects into zip top bags that were labeled with collection location, type of trap, and date of collection. Trapped insects were frozen initially, then placed into vials with 70% ethanol for storage. Bees were dried to restore pubescence before being pinned, databased, and labeled with collection information. Bees were identified to species using published keys [34,35,36,37,38,39] and reference material in the A. J. Cook Arthropod Research Collection (Michigan State University). Voucher specimens are deposited at Michigan State University.

#### 2.1.2. Statistical Analysis

Bee abundance was analyzed with a generalized nonlinear mixed effects model using the ‘glmer’ function in the ‘lme4’ package [40] in R version 3.2.2 [41]. The model was fit with a Poisson distribution after visual assessment of the count data. The total number of bees collected was analyzed in response to trap type and trap location, with site and date as random factors. Inclusion of both site and date accounted for some sites receiving fewer collection periods than others, and for non-independence between sites receiving two collection deployments. Significant main effects at α = 0.05 were followed by pairwise comparisons using Tukey’s HSD.

Non-metric multidimensional scaling was used to visualize similarities among bee communities for each trap type and field location, summed across all sites and dates. Bray-Curtis distances among communities were analyzed using the ‘adonis’ function in the ‘vegan’ package [42] in R. Goodness-of-fit was estimated with stress (S) and fit (adonis R) values. Species vectors were fitted to the NMDS using the ‘envfit’ function in the ‘vegan’ package [42]. Shannon-Weiner diversity indices were calculated for bee communities across trap types. An indicator species analysis was conducted to determine if any particular species were strongly associated with any trap type or trap location. Analysis was conducted using the ‘indval’ function in the ‘labdsv’ package [43], generating indicator values for each species.

To confirm that bee communities were sufficiently sampled, species accumulation curves were generated using the ‘specaccum’ function in the ‘vegan’ package [11]. Random resampling with 100 permutations estimated the number of species expected as sampling increased [44]. Species accumulation curves were generated for the entire experiment and for each trap type.

### 2.2. Bumblebee Potato Flower Visitation

#### 2.2.1. Data Collection

Potato (*Solanum tuberosum* L. var. Atlantic) plants were grown in the greenhouse at Michigan State University, East Lansing, MI from seed potatoes in 12 cm diameter plastic pots in a perlite soil mix (Suremix Perlite, Michigan Grower Products Inc., Galesburg, MI, USA). Plants were fertilized weekly with a 5 g/L 20-20-20 N-P-K (Scott’s Miracle-Grow Products, Inc., Marysville, OH, USA) solution. When plants first started emerging from the soil, each pot received 18.7 µL Admire Pro^®^ (imidacloprid, Bayer Crop Science, Monheim am Rhein, Germany; equivalent to the high label rate of 0.36 L/ha active ingredient) diluted in 20 mL distilled water. The experiment was replicated over two time periods (4 replications/period, N = 8) in the summer of 2016. Half of the plants received imidacloprid on 9 June 2016, and bee visitation observations were conducted between 11 July and 14 July 2016. Half of the plants in the second time period received imidacloprid on 24 June 2016, and visitation observations were conducted between 22 July and 27 July 2016. Plants that were starting to flower (10–20% open bloom) were moved from the greenhouse to outdoor enclosures at the Entomology Research Farm at Michigan State University, East Lansing, MI, USA. Four 1.8 × 1.8 × 1.8 m mesh enclosures each contained 10 plants (five imidacloprid-treated and five untreated plants placed randomly within the enclosure) and one bumblebee (*Bombus impatiens* Cresson, Hymenoptera: Apidae) colony. MINIPOL^®^ colony boxes were obtained from Koppert Biological Systems™ Inc., (Berkel en Rodenrijs, The Netherlands), shipped overnight from Howell, MI, USA. Each box contained one small colony of 10–15 individuals. Colony boxes were placed immediately into the enclosures upon arrival to Michigan State University and visitation observations began 2–5 days later when bees were acclimated to their new surroundings.

Observations were conducted each morning from 8 to 9 a.m. with one observer rotating among the four cages for one hour, counting bee visits to flowers and noting plant treatment. Duration of observations was limited to one minute per cage before moving to the next cage to limit counting the same bee multiple times. Observations were conducted during peak bloom (50–100% open bloom) and ceased when flowers began to drop. After the 1 h observation period, floral visit duration was recorded for 10–20 additional pollinator visits. On 14 July 2016, flowers and aboveground vegetative material were collected from two treated and two untreated plants per cage, dried, and weighed. Samples of flower and plant tissue were analyzed at Michigan State University for imidacloprid using the QuEChERS method [45].

#### 2.2.2. Statistical Analysis

Dry biomass of flowers per plant and plant tissue per plant, visitation frequency (total visits/hour for each trial, square-root transformed) and visitation duration (seconds/visit, log-transformed) were each analyzed with a linear mixed effects model, using the ‘lme’ function in the ‘nlme’ package [46] in R. Data transformation were performed so that distributions of residuals met the assumptions of linear models, confirmed by quantile-quantile plots. Models included imidacloprid treatment as a fixed factor, and date and enclosure (frequency analysis only) as random factors. For effects 0.5 > *p* > 0.1, a power analysis using the ‘pwr’ package [47] was performed to estimate the effect size needed to observe significant differences at α = 0.5 with our sample size.

## 3. Results

### 3.1. Bee Community Survey in Commercial Potato Fields

Fifty-eight species of bees from 16 genera and five families were captured in potato field traps (Table 2). *Lasioglossum* spp. (Halictidae) comprised 73% of all bees captured. Most bees in our samples were host plant generalists except for four species of the family Apidae: *Melissodes agilis* Cresson (specialist on *Helianthus* spp., Asteraceae), *M. desponsus* Smith (specialist on *Cirsium* spp., Asteraceae), *M. subillatus* LaBerge (specialist on Asteraceae), and *Peponapis pruinosa* (Say) (specialist on Cucurbitaceae, [48]). In addition to the European honeybee *Apis mellifera* L., we found two exotic, but naturalized species, *Lasioglossum zonulum* (Smith) and *L. leucozonium* (Schrank) [48], but all other sampled species are native in our area. Ground nesting bees comprised 74% of all species; we found just a few species that nest in cavities, plant stems, or rotting wood (Table 2). The proportion of eusocial (28 species) to solitary species (26 species) was about equal in our samples.

**Figure 3:**
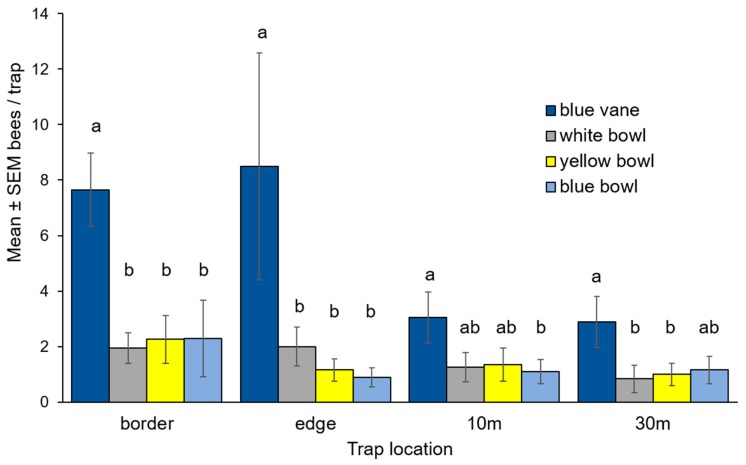
Mean ± SEM number of bees captured in commercial potato fields in Michigan in 2016 by trap type and trap location. Bowl traps were elevated on a platform at canopy height, with one bowl of each color on each platform. A transect of four bowl trap platforms ran from bordering vegetation roughly 10 m from the potato field (“border”), at the edge of the field (“edge”), 10 m into the potato field (“10 m”), and 30 m into the potato field (“30 m”). Blue vane traps were suspended from a metal pole at canopy height, and placed in a parallel transect 10 m from the bowl trap transect. Different letters above bars indicate significantly different (α = 0.05) abundances in Tukey’s HSD test within locations.

Out of the 756 bees we collected, 68% were found in traps placed either at the field edge or in the border outside the field (Table S2). There was an interactive effect of trap type and trap location (interaction: χ^2^ = 36.7, df = 9, *p* < 0.001; trap type: χ^2^ = 315.6, df = 3, *p* < 0.001; trap location: χ^2^ = 88.9, df = 4, *p* < 0.001). Blue vane traps caught almost four-times as many bees as the bowls, and this effect was greatest in the bordering vegetation and at the potato field edge (Figure 3 and Table S3). Blue vane traps captured 438 individuals representing 41 species. Blue bowl traps captured 103 individuals representing 27 species. White bowl traps captured 116 individuals representing 28 species. Yellow bowl traps captured 105 individuals representing 29 species (Table S3).

**Figure 4:**
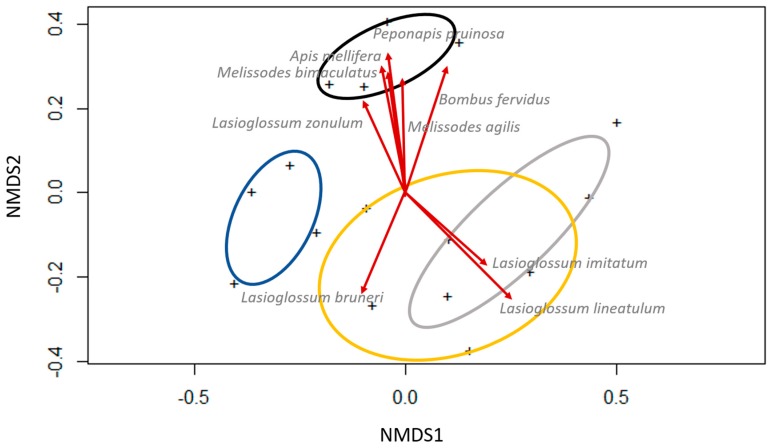
Visualization of bee communities by trap type in non-metric multidimensional ordination space. Bowl traps were elevated on a platform at canopy height, with one bowl of each color on each platform. A transect of four bowl trap platforms ran from bordering vegetation roughly 10 m from the potato field (“border”), at the edge of the field (“edge”), 10 m into the potato field (“10 m”) and 30 m into the potato field (“30 m”). Blue vane traps were suspended from a metal rod at canopy height, and placed in a parallel transect 10 m from the bowl trap transect. Bee communities (+) are the sum of individuals found in each trap type and location, summed across sites and dates. Ellipses represent 95% confidence intervals around communities defined by trap type: blue vane trap (black ellipse), blue bowl trap (blue ellipse), white bowl trap (grey ellipse), and yellow bowl trap (yellow ellipse). Bee communities did not differ across trap locations; 95% CIs for trap location are not presented. Red vectors are species with significant (*p* < 0.05) effects in the ‘adonis’ model (see text for values).

Bee communities did not differ across trap location within field (S = 0.2, R^2^ = 0.2, *F* = 1.1, df = 3, *p* = 0.4), but differed across trap types (S = 0.2, R^2^ = 0.3, *F* = 2.0, df = 3, *p* < 0.001). Species with significant NMDS scores were *Apis mellifera* (r^2^ = 0.5, *p* = 0.02), *Bombus fervidus* (Fabricius) (r^2^ = 0.6, *p* = 0.001), *Lasioglossum bruneri* (Crawford) (r^2^ = 0.4, *p* = 0.04), *L. imitatum* (Smith) (r^2^ = 0.4, *p* = 0.02), *L. lineatulum* (Crawford) (r^2^ = 0.8, *p* = 0.01), *L. zonulum* (r^2^ = 0.3, *p* = 0.05), *Melissodes agilis* (r^2^ = 0.5, *p* = 0.01), *M. bimaculatus* (Lepeletier) (r^2^ = 0.4, *p* = 0.004), and *Peponapis pruinosa* (r^2^ = 0.6, *p* = 0.003) (all species scores listed in Table S4). All of these species were associated with blue vane traps, except *L. bruneri*, *L. imitatum*, and *L. lineatulum*, which were associated with white and yellow bowl traps (Figure 4). Indicator species values showed that individual species were not significantly associated with any trap type or trap location (Holm-corrected, all species *p* > 0.07). Shannon-Weiner diversity indices were 2.7-2.9 for all trap types.

**Figure 5:**
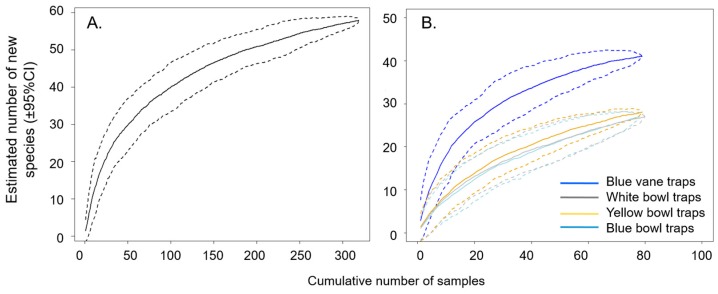
Species accumulation curves for bees surveyed in commercial potato fields in Michigan. Bees were collected in four different trap types at 12 field sites during the 2016 growing season. Species accumulation curve for all trap types pooled together (**A**) and by trap type (**B**). Dashed lines indicate 95% confidence intervals.

Species accumulation curves estimated approximately 60 bee species in these potato fields (Figure 5A); therefore, it seems unlikely that further sampling would add many new species. Bowls appear slower than blue vane traps in accumulating species (Figure 5B).

### 3.2. Bumblebee Potato Flower Visitation

**Figure 6:**
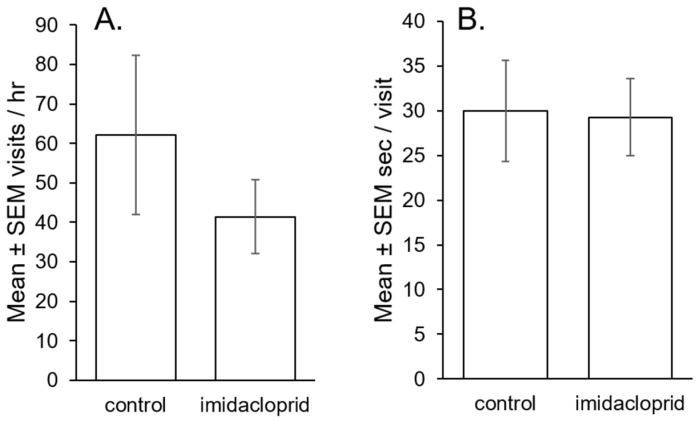
Mean ± SEM flower visits per hour (**A**) and seconds per visit (**B**) by *Bombus impatiens* bees in mesh field enclosures. Enclosures contained five imidacloprid-treated and five untreated flowering potato plants and a single bee colony of 10–15 foragers. Bees were allowed to forage and were observed for 15 min/enclosure/day.

Visitation frequency and duration were not statistically different between imidacloprid-treated and untreated flowers (*p* = 0.09, *p* = 0.8, respectively; Figure 6a,b). Power analysis estimated differences in visitation rates would need to be approximately five times greater to be significant at α = 0.05 with our sample size. Imidacloprid levels were 2.53 ± 0.62 µg/g in leaf tissue and 2.02 ± 0.71 μg/g in flower tissue of treated plants (mean ± SEM). Imidacloprid was not present in leaves or flowers of untreated control plants. The weight of vegetative tissue and floral tissue biomass were not different between imidacloprid-treated and untreated plants (all *p* > 0.08).

## 4. Discussion

In our community survey, 58 species of bees were found in or near Michigan potato fields, despite the relative lack of diverse resources in these monocultures. Since most of the trapped species are host generalists, it is likely that at least some of them visit potato flowers, although field visitation observations were not part of this study. Areas around potato fields likely provide nesting and foraging resources for many of these bees. Alternatively, the large flush of flowers offered by hectares of potatoes blooming at the same time may attract bees from distant areas to the field. For example, some bumblebees have a nearly 10 km foraging range [93].

Bumblebees are main pollinators of *Solanum* spp. in North America, but out of the seven *Bombus* species we found, *Bombus impatiens* is the only one that has been confirmed pollinating potato flowers [94]. Five bumblebee species we recorded were also found in surveys of New York potato fields, but these were associated with plants located near the fields [5]. *Bombus fervidus*, one of the more abundant bumblebees in our samples, was not attracted to potato flowers in a cage experiment [94]. As in our samples, honeybees have been observed in potato fields previously and may be initially interested in potato flowers, but due to the lack of nectar and the honeybees’ inability to buzz-pollinate, these bees are not considered pollinators of potatoes [94].

*Lasioglossum* was the most diverse genus with 25 species in our samples. These bees are typically polylectic and are known to visit *Solanum* spp. Although *Lasioglossum* and some other halictid bee genera we collected are known to buzz-pollinate [95,96], *Lasioglossum* may be primarily pollen scavengers of *Solanum* [97], collecting accessible pollen from the flower surface, but providing little pollination benefit. Their visitation to potato flowers is supported by the fact that 27% of *Lasioglossum* spp. were found in traps 10 and 30 m inside the potato field. In addition, their small body size should result in a short foraging range [98] that implies their ability to nest in and around potato fields. Two species of small *Sphecodes*, potentially cleptoparasites of *Lasioglossum*, were also found 10 m into the field. While *Lasioglossum* was the most diverse genus in our samples, their abundance and species richness responded negatively to pesticides in apples, indicating that these bees are sensitive to pesticide toxicity [99]. Therefore, further studies should explore the interactions of these bees with potatoes and the potential impact of pesticides on them.

Simplified landscapes of non-pollinator dependent crops constitute a substantial proportion of the Midwest US land cover and are blamed, in part, for declining bee populations [100]. Research investigating bee communities within non-pollinator dependent crops is rare; however recent studies in corn, soybean, biofuel crops (corn, switchgrass, mixed prairie species) [11,12,13,101,102], and now potato fields (this study) are improving understanding of bee diversity in non-pollinator dependent crops. Bee abundance and richness is typically lower in intensive crops relative to more diversified habitats [101], however a substantial number of bee species remain in these landscapes. An exhaustive comparison of non-pollinator dependent crops is difficult due to different sampling efforts and the extent of taxonomic resolution among studies, however some similarities are evident. Ground-nesting bees, such as *Melissodes* and *Lasioglossum* spp., dominate in these areas as expected given the limited opportunities for cavity-nesters in crop fields. Some stem-nesting species such as *Ceratina* and *Hylaeus* remain in low abundance relative to semi-natural areas, although they can be abundant in switchgrass biofuel crops [101]. The bulk of individuals are hyper-generalist foragers that visit flowers across many plant families, including *Melissodes bimaculatus*, *Agapostemon virescens*, and *Lasioglossum* spp., which dominate the few studies identifying bee species in non-pollinator-dependent crop systems [11,12,101,102]. *Melissodes bimaculatus*, honey bees, and halictid bees, in particular *Lasioglossum*, are known to use floral resources such as corn and grass pollen [103,104] that are typically avoided by other bees in the region.

Trap type, color, and height can influence wild bee captures [105,106,107,108]. Indeed, we captured mostly *Lasioglossum* spp. in bowl traps, but the only representatives of *Anthophora*, *Peponapis*, and some species of *Bombus* and *Melissodes* were collected in blue vane traps. In our study, large-bodied apid bees, such as *Bombus*, were collected in greater numbers in blue vane traps relative to bowl traps. Blue vane traps are effective at collecting bees, especially large-bodied Apidae, which is consistent with other studies that have employed them [31,105,107,108,109]. In fact, blue vane traps in a simple agricultural landscape (soybean) collected a surprising number of large-bodied apid bees, including oligoleges of non-crop flowers [11]. Oligolectic bees may appear to be positively associated with increasing area of field crops when blue vane traps are used [109]. One possible explanation is that UV-reflecting vane traps have greater relative attractiveness to bees flying in simple landscapes with few resources.

The third-most abundant species in our study, *Peponapis pruinosa*, is a cucurbit specialist [48], which does not visit potato for pollen but was collected in relatively high abundance in blue vane traps. The presence of oligolectic bees in non-host crop fields should not be interpreted as evidence that they use or persist in those crop fields. They may instead be far-flying species moving between sparse foraging resources that become attracted to the conspicuous traps deployed to capture them. Although oligolectic bees may be found in potato fields, the lack of nectar and suitable pollen rewards mean these bees are unlikely to forage on the neonicotinoid-treated potato plants.

The field survey allowed us to identify bee species associated with Michigan potato fields over the course of a field season, and our manipulative enclosure study provided preliminary insights to potential impacts of neonicotinoid-treated potatoes on the most common potato pollinator, the bumblebee [5]. Sublethal effects of neonicotinoids on bumblebee foraging have been observed [110], but in our study bumblebee visitation to potato flowers with or without neonicotinoid treatment was statistically similar. This may have been due to insufficient sample size, as our power analysis indicated that effect sizes would have had to be five times greater to see significant differences at our current sample size, or due to the fact that insecticide residues in pollen were low and had no measureable impact on foraging behavior. Since the hives were supplied with sugar water and potato flowers do not contain nectar, the only exposure bees had in our study to the neonicotinoid was through contact with potato flowers and ingestion of pollen. Although we did not specifically measure imidacloprid content in pollen, previous results indicate that low levels may have been the underlying cause of the lack of neonicotinoid effect on bees [17,24]. Field studies with neonicotinoid seed-treated crops, such as oil seed rape, sunflower, and corn, have also concluded that exposure to these crops poses low risks to bumblebees, even over several years [26,27,28,111].

Laboratory exposure to imidacloprid-laced food reduced subsequent bumblebee foraging efficiency on pollen, but not on nectar [110]. One hypothesis proposed to explain this was that the insecticide may have impaired the ability of bees to collect pollen [110]. Pollen is important for rearing young workers [112] and if bumblebees are feeding neonicotinoid-laced pollen to their brood, it may result in lower colony growth [21], but several field studies with bumblebees foraging on neonicotinoid-treated plants did not find negative impacts on their offspring [20,23]. Our results from the enclosures should be interpreted with caution, since we had a limited number of colonies (N = 8) and we did not mark bees individually.

## 5. Conclusions

Our study fills a knowledge gap by providing information on bees found in commercial potato fields. Since much previous attention has been focused on honeybees interacting with neonicotinoids, our contribution on bumblebees adds new details in this area. While further experiments with sufficiently larger sample sizes are needed to confirm our findings, our results are aligned with previous results indicating that many bee species found in potato cropping systems are also represented in other field crops in North America and that bumblebee exposure to imidacloprid-treated flowers did not have significant negative impacts on foraging behavior. Although here we did not measure other potential effects of imidacloprid on bumblebees, in the future, we need to investigate bumblebee colony responses to neonicotinoids under field conditions over several field seasons. This study will provide useful information for future research on the bee fauna in potato fields in other parts of the world.

## Figures and Tables

**Table 1:** Site descriptions of commercial potato fields used for bee surveys in 2016. Bees were surveyed with four white, yellow, and blue bowls and four blue vane traps at each site.

Site ID	Flower Color	Potato Variety	Latitude	Longitude
1	white	‘Pike’	43.4480	−84.8882
2	white	‘Pike’	43.4444	−84.8974
3	purple	‘Lamoka’	43.4444	−84.8974
4	purple	‘FL1922’	43.3209	−85.0123
5	white	‘Pike’	43.4480	−84.8882
6	purple	‘FL2137’	43.4106	−84.9183
7	pink	‘Lamoka’	43.3867	−84.9707
8	pink	‘Lamoka’	43.3369	−85.0547
9	purple	‘FL2137’	43.3956	−84.9047
10	white	‘Snowden’	43.3828	−84.9027
11	pink	‘Lamoka’	43.3722	−85.0747
12	purple	‘FL2137’	43.3564	−85.0493

**Table 2:** Species names and characteristics of bees captured in bowl and blue vane traps in 12 commercial potato fields in Michigan in 2016. Biological data are based on published studies and reviews [36,37,38,39,48,49,50,51,52,53,54,55,56,57,58,59,60,61,62,63,64,65,66,67,68,69,70,71,72,73,74,75,76,77,78,79,80,81,82,83,84,85,86,87,88,89,90].

Taxa	Nesting	Behavior	Native/Exotic	References	No. of Individuals	% of Total
ANDRENIDAE						
*Andrena miserabilis* Cresson 1872	ground	solitary	native	[74]	1	0.1
APIDAE						
*Anthophora abrupta* Say 1837	ground	solitary	native	[68]	1	0.1
*An. bomboides* Kirby 1837	ground	solitary	native	[67]	2	0.3
*An. terminalis* Cresson 1869	wood/cavity	solitary	native	[60]	6	0.8
*Apis mellifera* Linnaeus 1758	hive	adv. eusocial	exotic	[61]	24	3.2
*Bombus auricomus* (Robertson 1903)	hive	eusocial	native	[61]	1	0.1
*B. bimaculatus* Cresson 1863	hive	eusocial	native	[61]	7	0.9
*B. fervidus* (Fabricius 1798)	hive	eusocial	native	[61]	5	0.7
*B. griseocollis* (DeGeer 1773)	hive	eusocial	native	[61]	2	0.3
*B. impatiens* Cresson 1863	hive	eusocial	native	[61]	5	0.7
*B. perplexus* Cresson 1863	hive	eusocial	native	[61]	1	0.1
*B. ternarius* Say 1837	hive	eusocial	native	[61]	1	0.1
*Ceratina mikmaqi* Rehan and Sheffield 2011	stem	solitary	native	[82]	1	0.1
*Eucera hamata* (Bradley 1942)	ground	solitary	native	[65]	5	0.7
*Melissodes agilis* Cresson 1878 ^1^	ground	solitary	native	[36,89]	13	1.7
*M. bimaculatus* (Lepeletier 1825)	ground	solitary	native	[36,51]	13	1.7
*M. communis* Cresson 1878 ^2^	ground	solitary	native	[36]	3	0.4
*M. desponsus* Smith 1854 ^2,3^	ground	solitary	native	[36]	11	1.5
*M. subillatus* LaBerge 1961 ^2,4^	ground	solitary	native	[36]	2	0.3
*Peponapis pruinosa* (Say 1837) ^5^	ground	solitary	native	[48]	64	8.5
COLLETIDAE						
*Hylaeus affinis* (Smith 1853) ^2^	stem	solitary	native		3	0.4
*H. mesillae* (Cockerell 1896)	stem	solitary	native	[89]	1	0.1
HALICTIDAE						
*Augochlora pura* (Say 1837)	rotten wood	solitary	native	[80]	4	0.5
*Augochlorella aurata* (Smith 1853)	ground	solitary/eusocial	native	[66,69,72,73]	2	0.3
*Agapostemon texanus* Cresson 1872	ground	solitary	native	[55,77]	2	0.3
*Ag. virescens* (Fabricius 1775)	ground	communal	native	[85,86]	9	1.2
*Halictus confusus* Smith 1853	ground	solitary/eusocial	native	[76]	3	0.4
*H. ligatus* Say 1837	ground	eusocial	native	[70,75]	7	0.9
*H. rubicundus* (Christ 1791)	ground	solitary/eusocial	native	[79]	1	0.1
*Lasioglossum albipenne* (Robertson 1890) ^2^	ground	eusocial	native		2	0.3
*L. anomalum* (Robertson 1892) ^2^	ground	eusocial	native		1	0.1
*L. bruneri* (Crawford 1902) ^2^	ground	eusocial	native		13	1.7
*L. cinctipes* (Provancher 1888)	ground	eusocial	native	[72,73]	1	0.1
*L. coeruleum* (Robertson 1893)	rotten wood	eusocial	native	[81]	10	1.3
*L. coriaceum* (Smith 1853) ^2^	ground	solitary	native		12	1.6
*L. cressonii* (Robertson 1890) ^2^	rotten wood	eusocial	native	[39]	2	0.3
*L. ellisiae* (Sandhouse 1924) ^2^	ground	eusocial	native		3	0.4
*L. heterognathum* (Mitchell 1960) ^2^	ground	eusocial	native		4	0.5
*L. illinoense* (Robertson 1892) ^2^	ground	eusocial	native		13	1.7
*L. imitatum* (Smith 1853)	ground	eusocial	native	[64]	26	3.4
*L. laevissimum* (Smith 1853)	ground	eusocial	native	[71,72,73]	2	0.3
*L. leucocomum* (Lovell 1908) ^2^	ground	eusocial	native		48	6.4
*L. leucozonium* (Schrank 1781)	ground	solitary	exotic	[88,91]	67	8.9
*L. lineatulum* (Crawford 1906)	ground	eusocial	native	[56]	36	4.8
*L. macoupinense* (Robertson 1895) ^2^	ground	solitary	native		1	0.1
*L. oceanicum* (Cockerell 1916) ^2^	ground	eusocial	native		20	2.6
*L. paraforbesii* McGinley 1986 ^2^	ground	solitary	native		14	1.9
*L. pectorale* (Smith 1853) ^2^	ground	solitary	native		31	4.1
*L. perpunctatum* (Ellis 1913) ^2^	ground	eusocial	native		48	6.4
*L. pilosum* (Smith 1853) ^2^	ground	eusocial	native		157	20.8
*L. smilacinae* (Robertson 1897) ^2^	ground	eusocial	native		4	0.5
*L. versatum* (Robertson 1902)	ground	eusocial	native	[53]	11	1.5
*L. zephyrum* (Smith 1853)	ground	eusocial	native	[52]	1	0.1
*L. zonulum* (Smith 1848)	ground	solitary	exotic	[88,91]	24	3.2
*Sphecodes coronus* Mitchell 1956	ground	cleptoparasite	native	[62]	1	0.1
*S. cressonii* (Robertson 1903)	ground	cleptoparasite	native	[62]	1	0.1
*S. mandibularis* Cresson 1872	ground	cleptoparasite	native	[62]	1	0.1
MEGACHILIDAE						
*Megachile latimanus* Say 1823	ground	solitary	native	[87]	2	0.3

^1^ specialist on Helianthus; ^2^ nesting data based on consubgeners (e.g., [92]); ^3^ specialist on Cirsium; ^4^ specialist on Asteraceae; ^5^ specialist on Cucurbitaceae.

## Supplementary Materials

The following are available online at http://www.mdpi.com/2075-4450/8/1/30/s1, Table S1: Planting and insecticide application dates and rates for field sites; Table S2: The total number of bees by genus and trap location; Table S3: The total number of bees by genus and trap type; Table S4: Ordination scores of bee species; Figure S1: seasonal temperature and precipitation data of field study region.
